# Supplementary material for: Canadian Healthcare Professionals’ Views and Attitudes toward Risk-Stratified Breast Cancer Screening
Source: J Pers Med. 2023 Jun 21;13(7):1027. doi: 10.3390/jpm13071027 (PMC10381377; doi:10.3390/jpm13071027)
Supplement: Supplementary file 1 [file jpm-13-01027-s001.zip › jpm-2374205-supplementary.pdf]

Supplementary Material S1. Listing of Healthcare Professional Associations or Organisations who Disseminated the Study Invitation

Fédération des médecins omnipraticiens du Québec
Canadian Society of Breast Imaging
Association des radiologistes du Québec
CHU de Québec-Université Laval
Ordre des infirmières de recherche
Génome Québec
McGill Faculty of Medicine and Health Sciences Electronic Newsletter
McGill Department of Family Medicine Monthly Newsletter
Infolettre de Pulsar
Ordre des infirmières et infirmiers du Québec
Nurse Practitioners' Association of Ontario
Canadian Partnership Against Cancer
Canadian Association of Medical Oncologists
Canadian Association of Nurses in Oncology
BC College of Family Physicians
Registered Nurses Association of The Northwest Territories and Nunavut
Association of Regulated Nurses of Manitoba
Le collège des médecins de famille de Terre-Neuve-et-Labrador

Supplementary Material S2. Study Questionnaire

PERSPECTIVE
INTEGRATION & IMPLEMENTATION

Personalized breast cancer risk assessment and screening mammogram (PERSPECTIVE I & I)

Questionnaire for healthcare professionals

If you have any questions regarding this questionnaire, please contact us by email at:
info@etudeperspective.ca

About this questionnaire

- Researchers from University of Toronto, Université Laval, McGill University, and University College London have developed this questionnaire. It aims to gather the opinions, attitudes and expectations of healthcare professionals regarding a breast cancer screening approach based on an individual's risk of developing breast cancer in the future. We therefore invite you to answer this short questionnaire, which will take between **12 to 15 minutes**.

- **By completing this questionnaire, you consent to participate in this research.** The information you send will be kept strictly confidential and your identity will remain anonymous. **Please do not include any personal identifiable information (e.g. name).**

- This study is part of a larger project funded by Canadian Institutes for Health Research, Genome Canada, Genome Québec, Ontario Research Fund , the Quebec Breast Cancer Foundation as wells other partners and is aimed at understanding whether there is a benefit to women knowing their breast cancer risk and using this information to make an informed choice about breast cancer screening.

- There are **no right or wrong answers**, and we ask that you simply check off the answers that most apply to you. We encourage you to answer every question. If you come to a question that you do not want to answer, please skip that question and answer the remaining questions.

Supplementary Material S2. Study Questionnaire

Preamble:

Although screening has benefits such as reducing mortality through early detection, there are also potential harms including overdiagnosis (diagnosis and treatment of breast cancer that would never have been life-threatening). A risk-based breast cancer screening approach is being considered by the scientific community as an option to improve the benefit-harm balance of existing screening programs. This approach would have three stages, as shown in the diagram below:

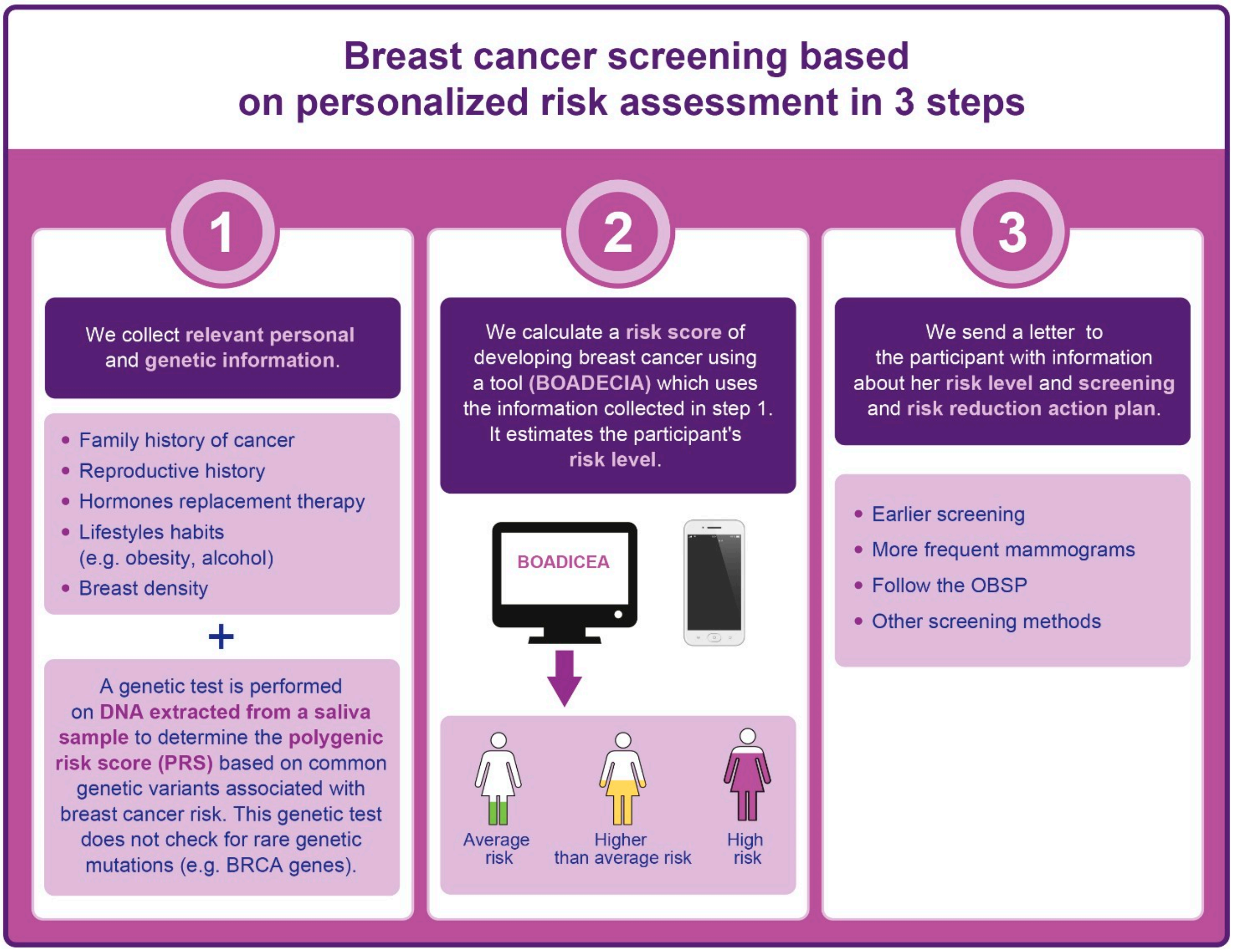

For more information about the project, visit the project website at: **www.cancercareontario.ca\breastriskstudy.**

Supplementary Material S2. Study Questionnaire

Q1. The Canadian Task Force on Preventive Health Care recommends that women at average risk aged 50-74 be screened with mammography every 2 years. Please, indicate how strongly you agree or disagree that breast screening is an effective method for early detection of breast cancer:

☐Strongly agree
☐Agree
☐Neither agree nor disagree
☐Disagree
☐Strongly disagree
☐Don't know

Q2. Have you ever heard of the Personalized Risk Assessment for Prevention and Early Detection of Breast Cancer: Integration and Implementation (PERSPECTIVE I&I) study? (Check all that apply)

☐I have never heard of it before today
☐I have heard about it in my clinical practice
☐I have heard about it through a website or social media
☐I have heard about it through a newsletter
☐I have attended a presentation about the study
☐I have at least one patient in the study
☐Other (please specify): ____________________________

Q3. Breast cancer risk assessment proposed in PERSPECTIVE I&I is based, among other factors, on a <u>polygenic risk score (PRS),</u> measured from a few hundreds of common breast cancer susceptibility genetic variants [single-nucleotide polymorphisms (SNPs)]. How familiar are you with the concept of PRS?

☐Very familiar
☐Familiar
☐Unfamiliar
☐Very unfamiliar
☐I don't know this concept

Q4. Breast cancer screening based on personalized risk assessment aims to adapt screening recommendations as a function of individual risk. Please indicate how strongly you agree or disagree with the following recommendations:

**Recommendations**	**Strongly agree**	**Agree**	**Neither agree or disagree**	**Disagree**	**Strongly disagree**
For women who are at **high risk** of breast cancer, **increase the frequency** of breast screening	☐	☐	☐	☐	☐
For women who are at **high risk** of breast cancer, **start the breast screening at an earlier age**	☐	☐	☐	☐	☐
For women who are at **higher than average risk** of breast cancer, **increase the frequency** of breast screening	☐	☐	☐	☐	☐
For women who are at **lower than average risk** of breast cancer, **decrease the frequency** of breast screening	☐	☐	☐	☐	☐
For women who are at **much lower than average risk** of breast cancer**, delay the start of breast cancer screening**	☐	☐	☐	☐	☐
For women who are at **much lower than average risk** of breast cancer, **do not offer** breast screening	☐	☐	☐	☐	☐

Q5a. <u>Women's risk of developing breast cancer will be estimated using several individual factors</u> such as family history of cancer, breast density, results of a genetic test to measure the polygenic risk score (PRS) and other risk factors. Please read the following statements and indicate whether you think this is a part of your scope of practice (check yes or no):

**Activities**	**Under the scope of my practice**
Discuss the advantages and limitations of personalized breast cancer risk assessment	☐Yes<br>☐No
Collect patient information required to perform a breast cancer risk assessment	☐Yes<br>☐No
Discuss the results of a breast cancer risk assessment with a patient	☐Yes<br>☐No
Explain to your patients the difference between the risk of developing breast cancer in the future and having a diagnosis of breast cancer	☐Yes<br>☐No

5b. Indicate how comfortable you would be based on your current knowledge to perform these activities with your patients:

Activities	Very comfortable	Comfortable	Neither comfortable or uncomfortable	Uncomfortable	Very uncomfortable
Discuss the advantages and limitations of personalized breast cancer risk assessment	☐	☐	☐	☐	☐
Collect patient information required to perform a breast cancer risk assessment	☐	☐	☐	☐	☐
Discuss the results of a breast cancer risk assessment with a patient	☐	☐	☐	☐	☐
Explain to your patients the difference between the risk of developing breast cancer in the future and having a diagnosis of breast cancer	☐	☐	☐	☐	☐

Q6. In your opinion, what 3 (three) spects of the Ontario healthcare system should be enhanced to implement breast cancer screening based on personalized risk assessment? (Check the three most important aspects in your opinion).

☐Number of primary care physicians
☐Number of nurse practitioners
☐Number of genetic counsellors
☐Number of geneticists
☐Remuneration of healthcare professionals
☐Medical training
☐Time allocated to a patient-physician appointment
☐Time allocated to a patient-nurse practitioner appointment
☐Access to a primary care physician
☐Access to a nurse or nurse practitioner
☐Access to breast screening (e.g. mammogram, MRI)
☐Other (please specify): ________________
☐None, I believe the healthcare system is ready

Q7. In your opinion, which healthcare professionals should play a role if breast cancer screening based on personalized risk assessment was implemented? (Check all that apply)

☐Primary care physician
☐Radiologist
☐Surgeon
☐Oncologist
☐ Nurse practitioner
☐Geneticist
☐ Genetic counsellor
☐Nurse navigator
☐Other (please specify): ______________

Q8. Based on your knowledge, what is your opinion regarding breast cancer screening based on personalized risk assessment, including risk calculation and interpretation? Please, indicate how strongly you agree or disagree with the following statements:

**Statements**	**Strongly agree**	**Agree**	**Neither agree, or disagree**	**Disagree**	**Strongly disagree**
I have enough knowledge regarding personalized breast cancer risk assessment	☐	☐	☐	☐	☐
I require more training on personalized breast cancer risk assessment	☐	☐	☐	☐	☐
I have time to educate myself on personalized breast cancer risk assessment	☐	☐	☐	☐	☐
I think it would be important to include more education on risk assessment, including genetic factors, in the medical curriculum	☐	☐	☐	☐	☐
I think it would be important to include more education on risk assessment, including genetic factors, in the nursing curriculum	☐	☐	☐	☐	☐

Q9. When you have general questions related to your clinical practice, what resources do you prefer to use? (Check all that apply)
☐Scientific publications
☐Training (courses, online conference or in-person)
☐Internet search engines
☐Colleagues
☐Government agencies
☐Other (please specify): ______________

Q10. For learning more about breast cancer screening based on personalized risk assessment, please select the three resource formats you find most useful for your clinical practice:
☐In-person training such as workshops
☐Online courses
☐Webinar type conference
☐Consultations with a geneticist or a genetic counselor
☐An application for your phone or tablet
☐Printed material
☐Website
☐Other (please specify): ______________

Q11. Concerning the resources in the previous question, what type of information would you like to find for your clinical practice? (Check all that apply)
☐General information on genetics
☐Information on common genetic variants (SNPs)
☐Information on the basics of personalized breast cancer risk assessment
☐Information on the calculation of a polygenic risk score (PRS)
☐Information on interpreting results of breast cancer risk assessment
☐Information on the best practices of breast cancer risk level communication
☐Information on breast cancer prevention
☐Information on the main ethical, legal and social challenges of personalized breast cancer risk assessment
☐Other (please specify): ______________

Q12. What is your gender?
☐Female
☐Male
☐Other
☐Prefer not to answer

Q13. What is your profession?

☐Physician
☐Nurse practitioner
☐Nurse navigator
☐Genetic Counsellor
☐Other (please specify): ______________

Q14. What is your main medical specialty?

☐Family medicine/ Primary care
☐Geriatrics
☐Genetics
☐Internal medicine
☐Public health medicine
☐Surgery
☐Radiology
☐Medical oncology
☐Surgical oncology
☐Obstetrics - Gynecology
☐Gynecologic Oncology
☐Other (please specify): _____________

Q15. For how long have you been practicing your profession?

☐Less than 5 years
☐Between 5 and 9 years
☐Between 10 and 14 years
☐Between 15 and 19 years
☐Between 20 and 25 years
☐More than 25 years

Q16. What is your main institution of practice?

☐Academic hospital
☐Community hospital
☐Family health team (Family Health Organizations (FHOs), Family Health Networks (FHNs))
☐Family Health Group (FHGs)
☐Community Health Centre (CHCs)
☐Comprehensive Care Models (CCMs)
☐Nurse practitioner-led clinic (NPLCs)
☐Private clinic
☐Other (please specify): _____________

Q17. In which Provinces or Territories do you mainly practice?

☐ Ontario
☐ Québec
☐ British Columbia
☐ Alberta
☐ Manitoba
☐ Saskatchewan
☐ Prince Edward Island
☐ New Brunswick
☐ Nova Scotia
☐ Newfoundland and Labrador
☐ Northwest Territories
☐ Yukon
☐ Nunavut

Do you have any comments or suggestions?

Thank you for your participation!

Supplementary Material S3. Healthcare professionals' attitude towards their scope of practice according to sociodemographic and professional variables

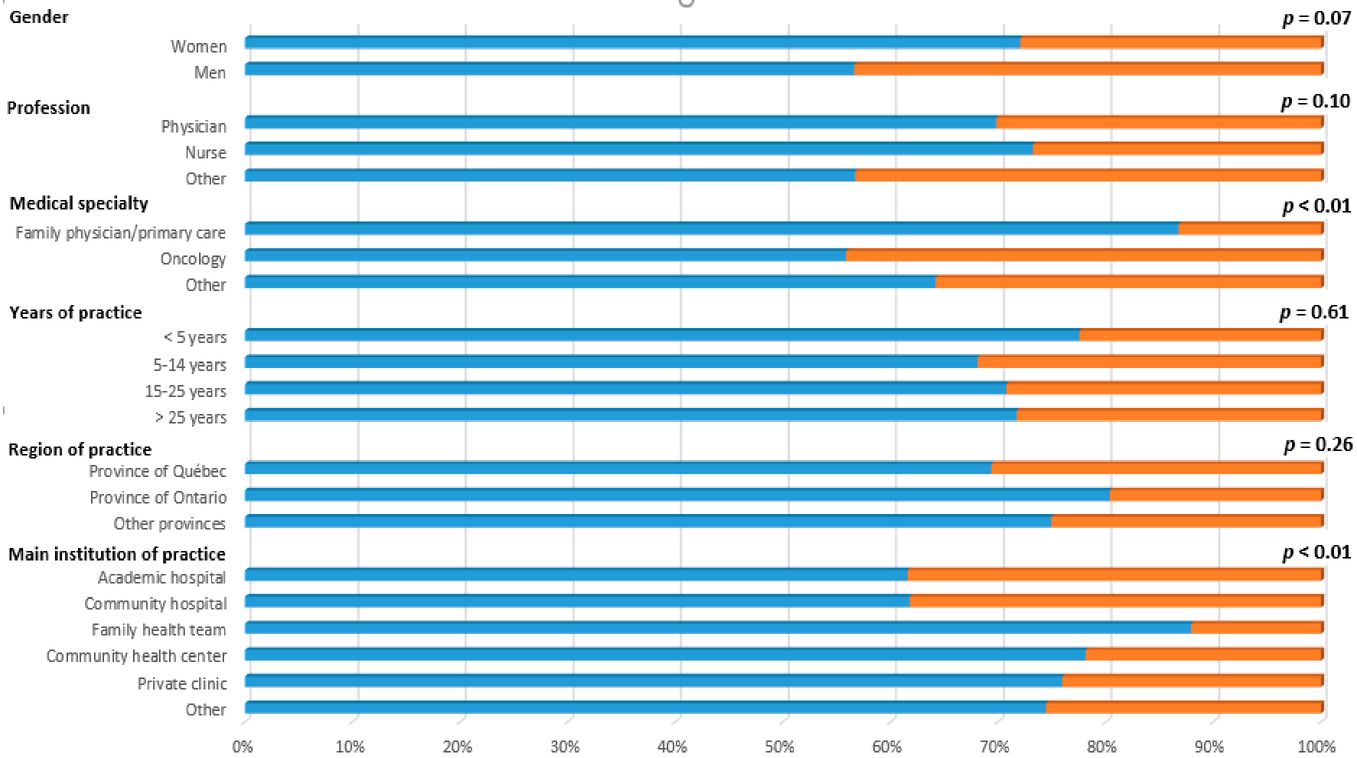

a1. Is «discussing the advantages and limitations of personalized breast cancer risk assessment» part of health professionals' scope of practice?

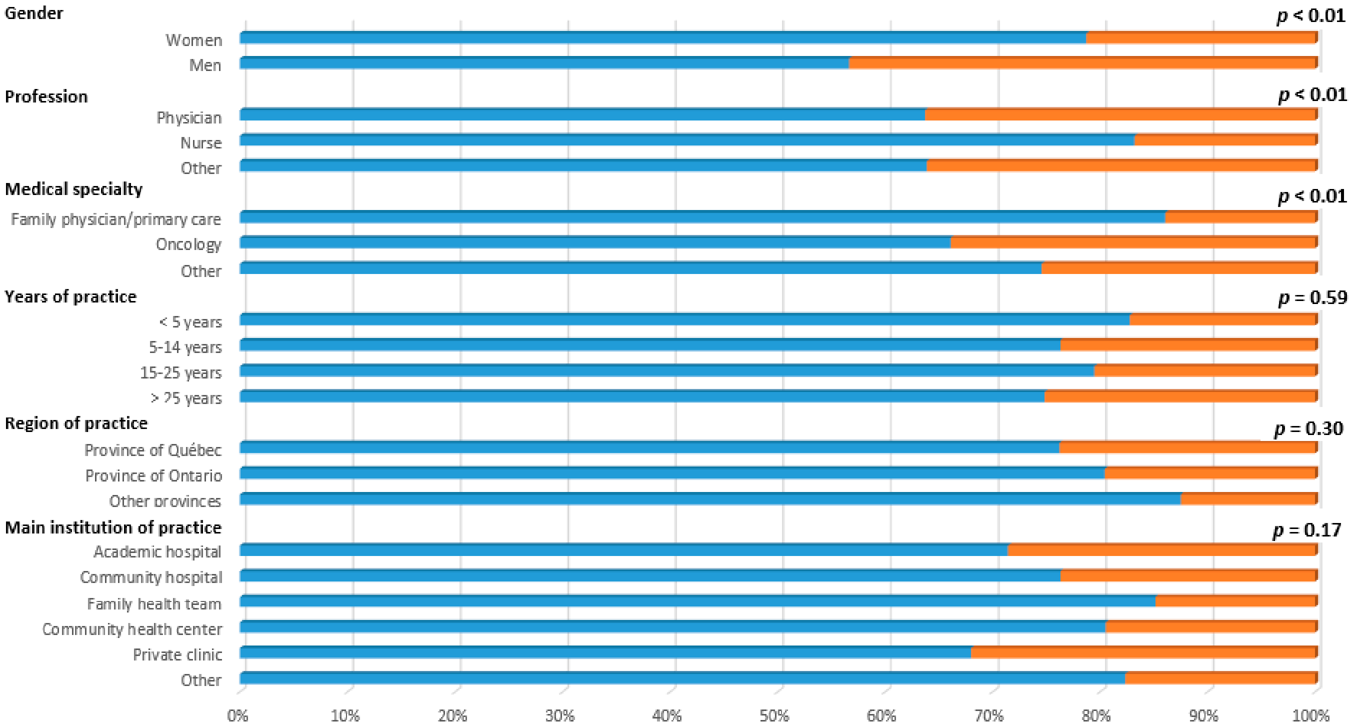

a2. Is «collecting patient information required to perform a breast cancer risk assessment» part of health professionals' scope of practice?

Supplementary Material S3. Healthcare professionals' attitude towards their scope of practice according to sociodemographic and professional variables

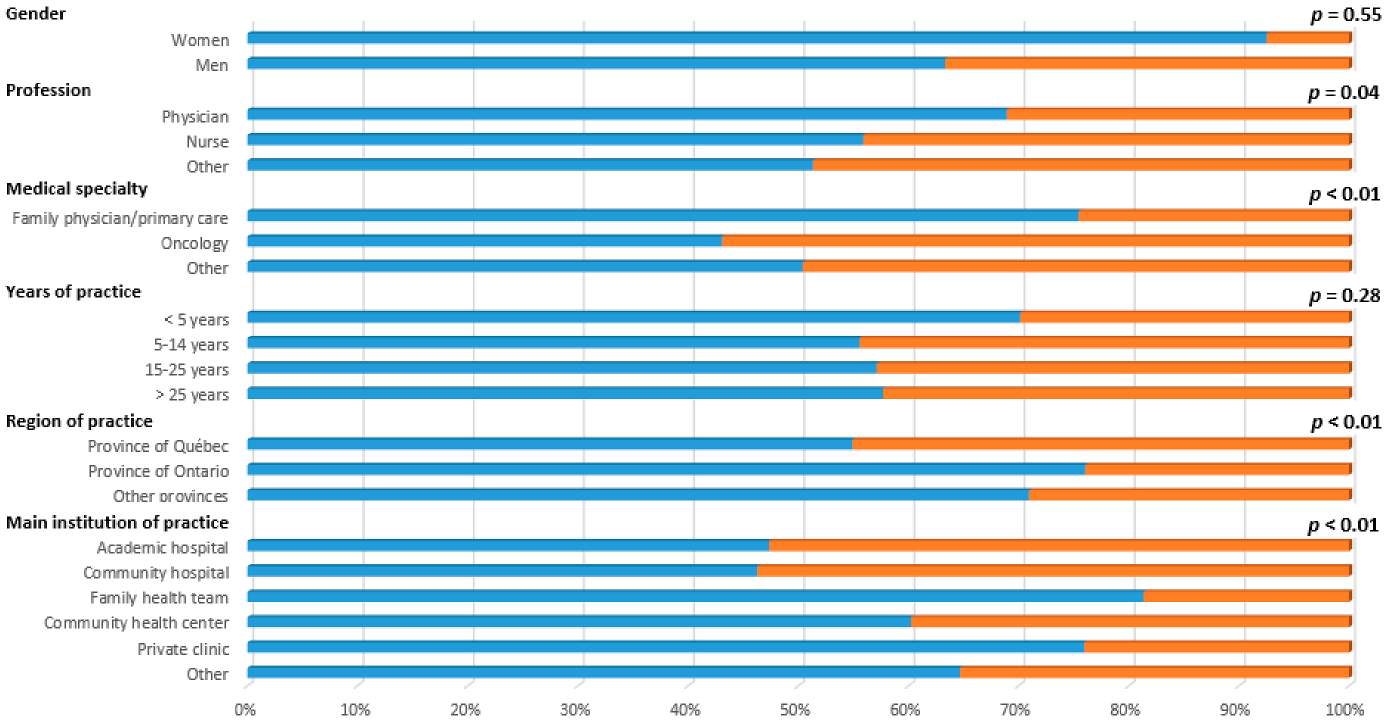

a3. Is «discussing the results of a breast cancer risk assessment with a patient» part of health professionals' scope of practice?

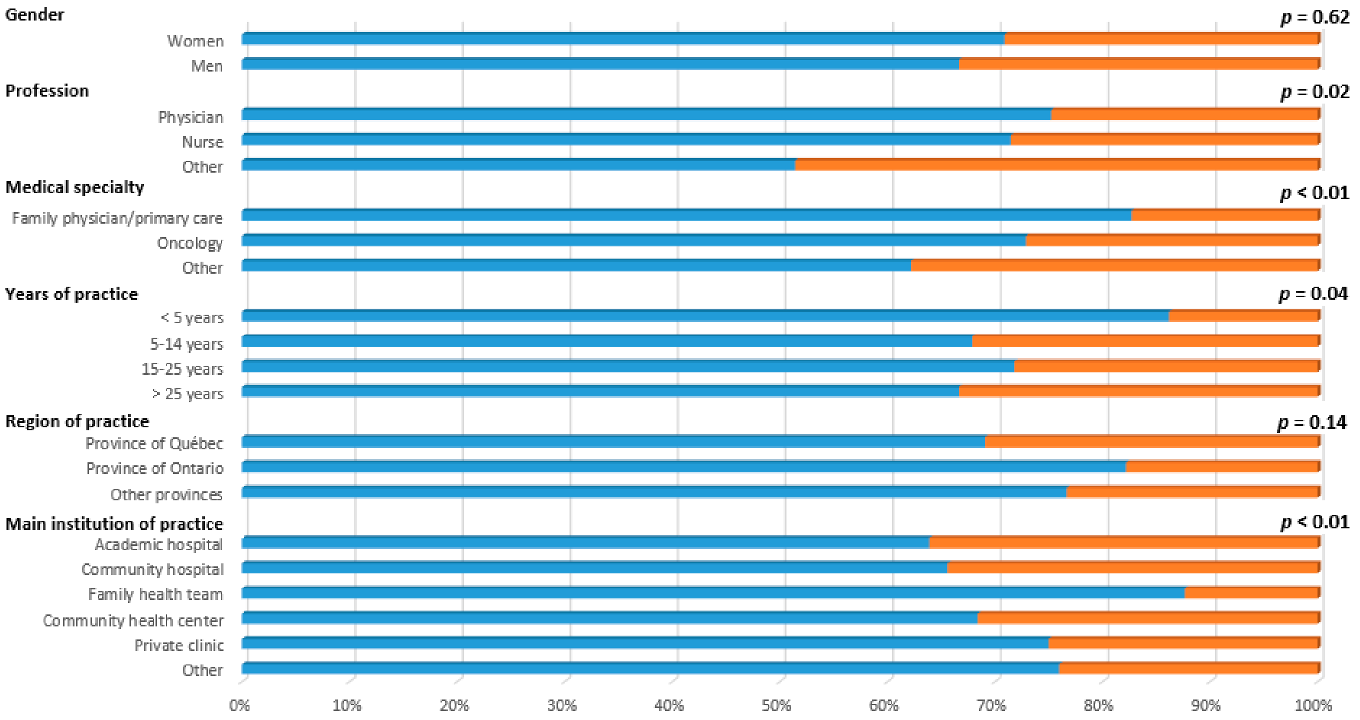

a4. Is « explaining to your patients the difference between the risk of developing breast cancer in the future and having a diagnosis of breast cancer» part of health professionals' scope of practice?

Supplementary Material S3. Healthcare professionals' attitude towards their scope of practice according to sociodemographic and professional variables

Legend: ■ Yes ■ No

Supplementary Material S4. Healthcare professionals’ attitude towards their comfort in the integration of the different roles according to sociodemographic and professional variables

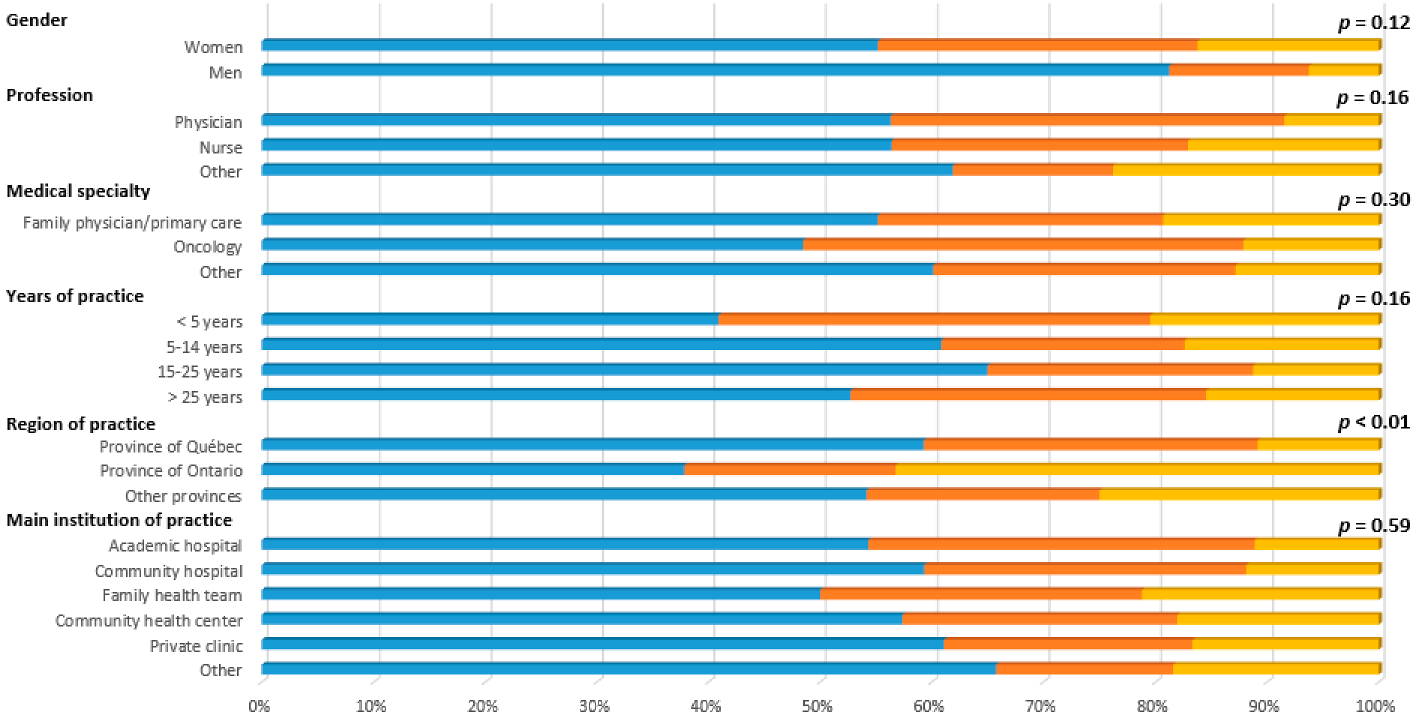

b1. Health professionals' level of comfort on «discussing the advantages and limitations of personalized breast cancer risk assessment»

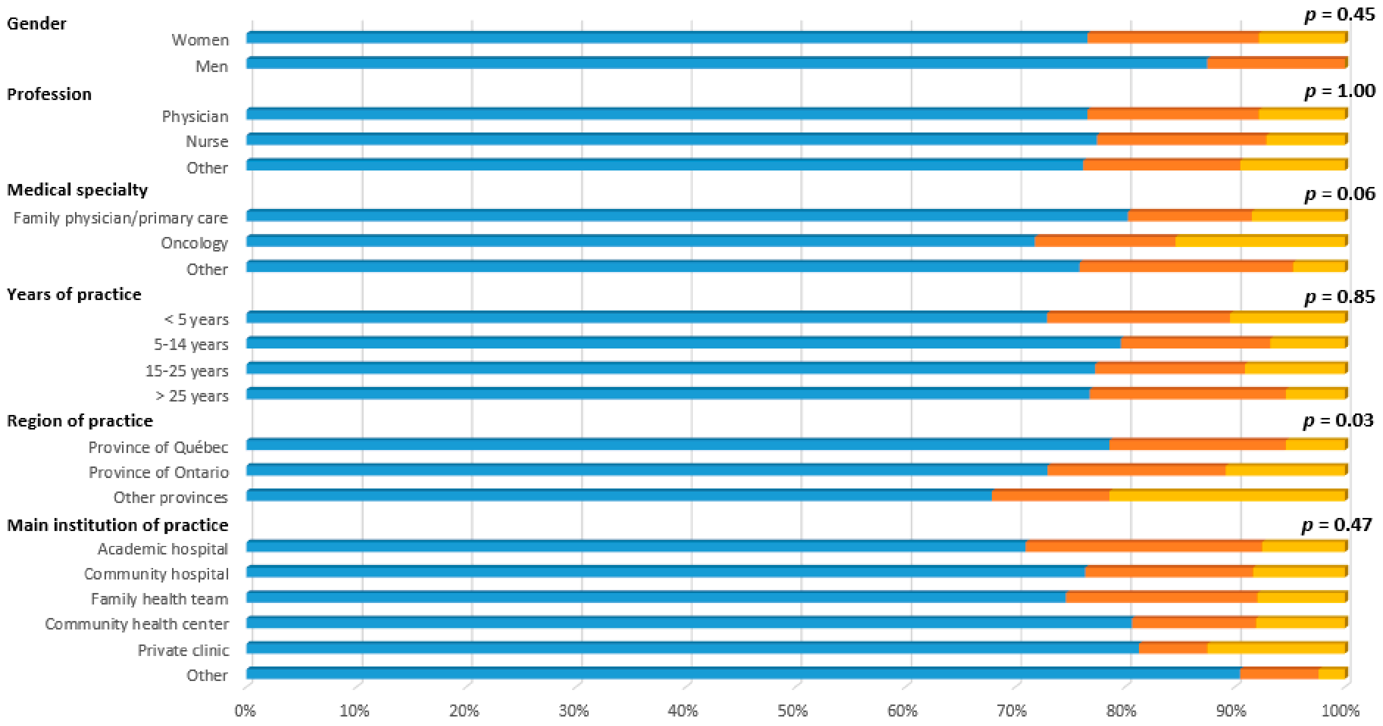

b2. Health professionals' level of comfort on «collecting patient information required to perform a breast cancer risk assessment»

Supplementary Material S4. Healthcare professionals' attitude towards their comfort in the integration of the different roles according to sociodemographic and professional variables

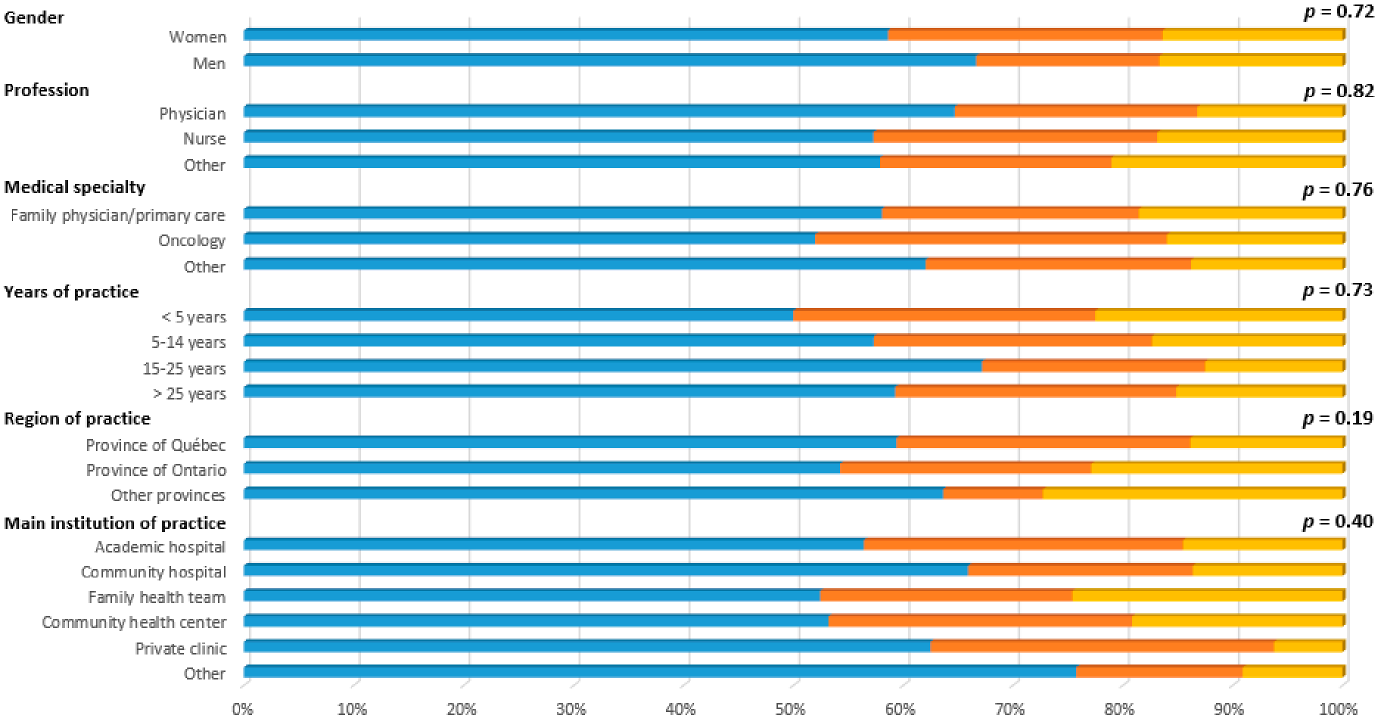

b3. Health professionals' level of comfort on «discussing the results of a breast cancer risk assessment with a patient»

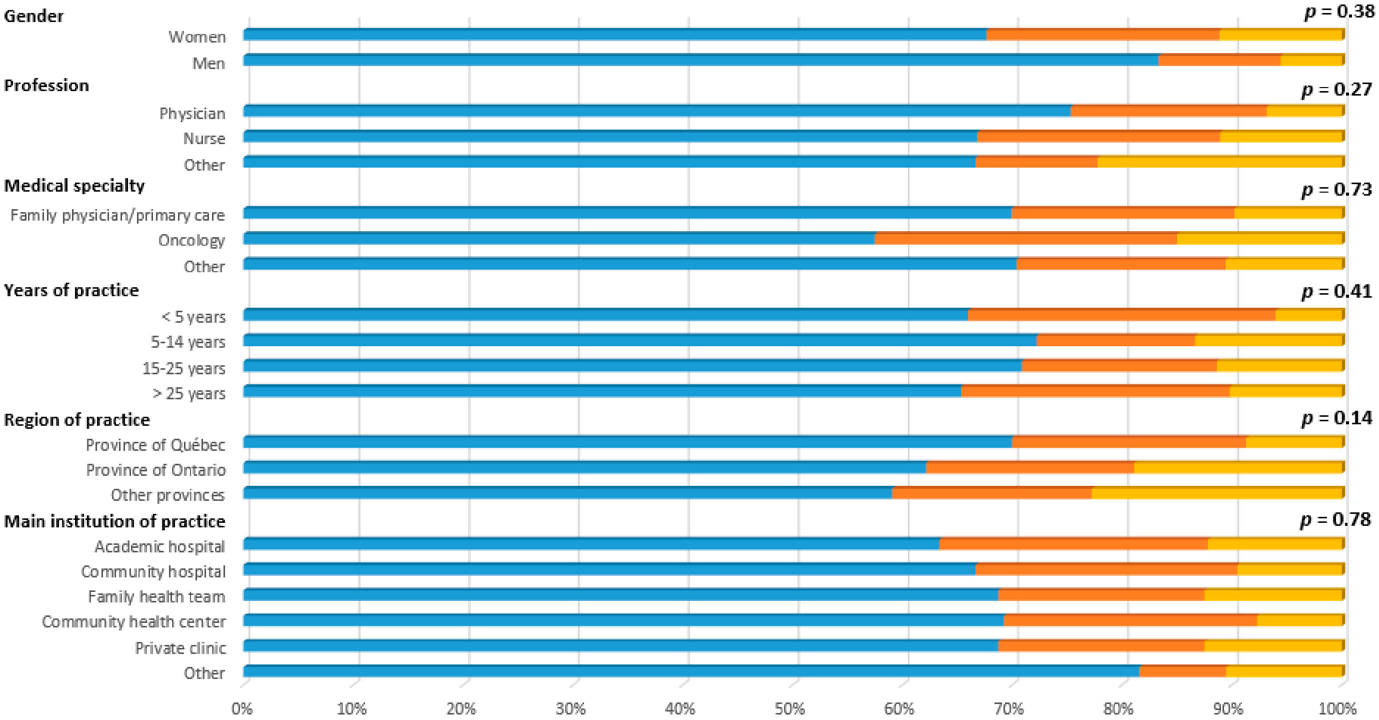

b4. Health professionals' level of comfort on « explaining to your patients the difference between the risk of developing breast cancer in the future and having a diagnosis of breast cancer»

Legend: Comfortable to very comfortable Neither comfortable nor uncomfortable Not very comfortable to uncomfortable

Supplementary Material S5. Synthesis of comments and suggestions to the open-ended question

Comments and suggestions received from respondents in the open-ended question

	**Demonstrate support toward risk-stratified BC**	**Synthesis of comments and suggestions**
**French Questionnaire**		
- Un sujet qui mérite d'être connu et diffusé.	1	
- Peut-être que vous devriez diminuer les critères d'emploi des infirmières conseillères en génétique au BAC au lieu de la Maîtrise afin d'augmenter le nombre de conseillères en génétique possibles. Fournir également des cours payés en anglais pour les infirmières en génétique.		Revise nurses' education requirement so that it is easier to become specialized in genetics
- Que le dépistage ne soit plus systématique mais personnalisé est une bonne idée, mais l'évaluation du risque ne peut pas reposer encore une fois sur les médecins de famille. Certaines personnes n'ont pas de MD de famille et les médecins de familles offrent déjà des soins de première, de deuxième et même de 3e ligne. Toutes les spécialités déchargent leurs suivis aux médecins de famille. Ajouter un calcul de risque aux médecins de famille revient à assumer que certaines n'auront pas ce calcul par manque de temps, parce d'autres problèmes de santé à discuter ou tout simplement parce que pas de médecin de famille. Des femmes alors à risque peut-être élevé pourraient se retrouver sans dépistage...	1	Consider the practical/logistic barriers of integrating this approach in primary care settings
- Je crois qu'un travail de collaboration avec md famille et IPSPL serait idéal. Le système actuel est tellement chargé par contre que l'introduction de nouvelles tâches nécessite de faire des choix, le temps étant une donnée importante dans l'équation.		Suggest to work on a collaboration between professionals

- Moderniser le PQDCS pour évaluer le risque des patientes par une collecte de données préalables pourrait être intéressant, systématique et simple. Une rencontre avec le médecin /IPSPL traitante suivant la réception du score de risque serait ensuite recommandée pour décider du choix de plan de dépistage en décision partagée.		Integrate within the provincial program
- bravo!	1	
- J'aimerais avoir des ressources pour pouvoir m'informer au moins de la base car il y a des patientes qui m'appellent des fois et cela serait pratique d'être au courant des notions de base en génétique. Pour le moment, je me réfère à ma collègue secrétaire qui travaille en génétique. Merci et bonne journée.		Provide basic genetic training to administrative staff
- La décision partagée devrait être centrale dans ce programme modifié. L'approche selon le risque est intéressante mais devra faire l'objet d'essais randomisés pour bien en évaluer les risques et les bénéfices. La décision partagée doit dans tous les cas, être mise au cœur des discussions, ce qui est loin d'être le cas actuellement.		Make sure shared decision making is central to the process
- Quelle bonne nouvelle que l'arrivée d'un dépistage personnalisé en cancer du sein! Bravo!	1	
- Faire des affiches à mettre dans les salles d'attente car les patients seront sensibilisées et pourront initier une discussion avec leur médecin Faire un dépliants à remettre a les patientes pour lecture et les patientes peuvent revenir sur le sujet à leurs prochains RDV distribuer à grande échelle de l'information par le gouvernement du Québec pour la sensibilisation et éducation sur les facteurs de risques du cancer du sein et importance de parler des ATCD familiaux ou autres aux médecins		Have mulitipronged patient and public information campaign
- Super intéressant!	1	
- Bonne chance. J'aimerais vraiment un Calculateur de risque automatisé dans une App (comme pour Tirads). Calculateur de risque du cancer du sein déjà existant avec différentes bases de données.		Provide access to an online risk calculator tool

Supplementary Material S5. Synthesis of comments and suggestions to the open-ended question

- Quelle est la part de la responsabilité sociale (RS) telle que définie par l'OMS ? est-elle explicite au sein du consortium de recherche dans la préparation et la réalisation de cette "évaluation personnalisée du risque de cancer du sein et dépistage par mammographie ? Existe-t-il un questionnaire similaire destiné 1) aux institutions académiques de professions de santé, 2) aux gestionnaires de systèmes de santé, 3) aux personnes décidant des politiques de santé, 4) aux communautés/personnes cibles ?		Make sure all societal aspects of implementing this approach are considered
- Droit être bien structuré avec l'implication dès le départ un groupe d'expert radiologues WG de la CAR (association canadienne des radiologistes) pour la validation des normes Implication aussi de l' ARQ pour le déploiement et les enjeux reliés à la modification du PQDCS		Involve experts in a carefully planned implementation of the approach
- Il ne faut pas implanter un tel dépistage sans prouver son efficacité		Make sure to prove that this approach is efficient and effective
- Très hâte que ceci soit applicable. Besoins cliniques déjà ressentis.	1	
- Avoir plus d'information sur les différents types de cancer durant la formation infirmière.		Enhance nurses' training
- Merci de nous donner l'opportunité de vous donner notre avis sur ce sujet très pertinent!	1	
- Je crois qu'aux États-Unis les mammos commencent 5 ans plutôt.		Compare with recommendations and standards from other countries
- Complètement d'accord avec l'initiative.	1	
- Très beau projet. Bravo!	1	
- Merci au nom de toutes les femmes!	1	

Supplementary Material S5. Synthesis of comments and suggestions to the open-ended question

- Si ce type de dépistage permet de rejoindre plus de femmes et de cibler celles qui sont plus à risque je pense que ce programme est pertinent	1	
- Une infirmière clinicienne spécialisée dans la santé des femmes pourrait être en mesure de gérer ce type de projet.		Nurses could be involved if access to proper training
- Très intéressant. Bien hâte d'avoir la suite.	1	
- super projet. Très bonne idée. Je crois que les infirmières GMF ont une très belle place. Plusieurs femmes redoutent la mammographie et je suis convaincue qu'avec un bon dépistage, nous attraperons des femmes qui n'auraient pas fait de mammo du tout.	1	Nurses could be involved if access to proper training
- Je trouve que c'est une excellente idée d'augmenter la prévention en fonction du risque. L'on devrait privilégier d'avantage la prévention en santé. Elle est souvent délaissée car, elle ne se chiffre pas aux yeux du gouvernement. De plus, la prévention est beaucoup moins couteuse à long terme.	1	Action in primary prevention should also be taken
- Plus de formations et de renseignements où chercher les informations concernant risques vs bénéfices		Need more information about the risks and benefits
- Sujet intéressant	1	
- Je suis contente d'avoir participé. On aimerait mieux être outillé avec des formations quel que soit le domaine soit gériatrie et autre pour pouvoir être en mesure de faire une évaluation de base pour le cancer du sein. En hébergement il y a aussi de jeunes résidentes, malheureusement on n'a jamais eu de formation sur le cancer du sein.		Need more information about the risks and benefits
- Je proposerais une clinique de dépistage où l'infirmière serait l'intervenante principale avec des médecins répondants.		Nurses should play a primary role in this approach

- Le dépistage du cancer c'est bien mais il ne faut pas faire peur aux gens. Aussi, si une personne considérée à très faible risque de cancer ressent des signes symptômes, il ne faut pas fermer la porte à un dépistage. Je n'aime pas votre approche protocolaire. Si quelqu'un n'entre pas dans le moule, vous ferez quoi?	Make sure to address this approach shortcomings and risks such as creating anxiety for women at high risk or denying services for women at very low risk	
- Si le gouvernement souhaite que l'on fasse le dépistage précoce du cancer du sein en dehors des CISSS-CIUSSS, il faut que de bons outils nous soient fournis pour être bien encadré. Ne pas sortir une nouvelle procédure bancale qui peut être fait pas n'importe qui, n'importe où. Avoir accès aux ressources par la suite du diagnostic pour les patients qui score haut. Qu'il y ait un corridor de soin clair et simple pour les patients qui ont besoin de plus d'examen et/ou suivi.	Implementation process should be well thought off	Access to resources
- Augmenter la bourde auprès des étudiants en IPSPL	Enhance nurses' training	
- Il semble manquer la place des infirmières cliniciennes. On fait de l'enseignement ++ et explications aux pts que le MD n'a pas détaillés. On décortique afin de rassurer apaiser les patients et familles :)	Nurses should play a primary role in this approach	
- IL SERAIT UTILE DE FORMER LES SUPER INFIRMIÈRES POUR DÉBUTER LA PRÉVENTION ET DONNER INFOS AUPRÈS DE LA CLIENTÈLE	Nurses should play a primary role in this approach	
- Le système actuel en place est bien, mais possède beaucoup de failles. Entre autre, les suivis et les oublis de suivis. Un outil ou un système de suivi serait apprécié par les patientes, qui sont souvent très inquiètes d'avoir été mise de côté par mégarde.	Implement a systematic follow up tool	

Supplementary Material S5. Synthesis of comments and suggestions to the open-ended question

- Nous faire un suivi de la présente évaluation et des retombés.		Interest in the results of our research project	
- Des informations sur l'influence du moment où l'âge du test. Savoir si l'allaitement a une influence sur le changement des prévisions. À quelle fréquence les prévisions doivent être réévaluées.		Provide more information about the approach	
- Une formation de soir accessible à domicile serait bienvenue		Access to an online training	
- Je trouve que c'est une bonne initiative de développer une évaluation personnalisée de ce risque et par conséquent adapter le dépistage en fonction de chaque personne et de son degré de risque, plutôt que de s'en tenir uniquement au plan de dépistage général du cancer du sein en l'appliquant de la même façon a tout le monde.	1		
- Meilleure formation ajout d'infirmières Praticiennes Spécialisées pour évaluer ces femmes. Dépistage à commencer à 40 ans ou avant si test génétique disponible pour évaluer le risque de développer un cancer. Démystifier la mammographie....		Provide more detailed information about the approach	Nurses should play a primary role in this approach
- Un grand MERCI pour vos recherches. Je parle en tant que professionnelle, en tant que mère aussi. Merci infiniment, continuez votre beau travail de recherche, on a besoin de vous!	1		
- J'aimerais en apprendre davantage à ce sujet. Très intéressant.	1		
- Merci	1		
- Excellente idée d'impliquer des professionnels de la santé directement touchés par la venue de cet implantation	1		
- Je trouve les infos très intéressantes. Cependant je faillis ne pas répondre croyant au prime à bord qu'il s'agissait d'un pourriel. Un petit texte plus explicite accompagnant le sondage mettrait les destinataires en confiance J'ai dû appeler pour m'assurer que c’est serieux. Merci et bonne continuation.	1		

- Si formation dans le futur, aborder les avantages et inconvénients en lien avec tout ça et impact psychologique sur les clientes qui ont un résultat à haut risque. Le surdiagnostic.		Need more information about the risks and benefits
- Très intéressant comme nouvelle pratique, personnaliser la prévention.	1	
**English version of the Questionnaire**		
- Am very glad that there is enlightened exploration of routine breast screening practices with risk stratification. I personally have always challenged the recommendations and practices. We also need improved technology to assess breast health, as all breast sizes and density are not equal. There are insufficient vertical MRIs available for routine screenings. I eagerly look forward to improved risk assessment and decision making supports.	1	Improve access to breast screening technology and developped better technology
- utilize the NP role		Nurses should play a primary role in this approach
- Patient themselves should be able to initiate a breast cancer screening at anytime. No one should have the ability to cancel or deny screening ever. Women know their breasts. Booking/ techs should never be able to cancel an appointment for screening or any screening whatsoever if a patient themselves have concerns		Make sure patient have an active role and that this approach is patient-centered
- This sounds very good. Women need to know their lifetime risk of developing breast cancer and their risk of dying from breast cancer. I believe many overestimate.	1	
- It will be nice if it's like Framingham risk score for CAD calculator I believe current recommendations are not the best Thank you for working on this!!	1	Should compare to other risk score
- Is there a web site where I can review Perspective I&I now?		Need more information about the risks and benefits

- Animated learnings, videos, interactive CME not passive, game-based learning, app		Access to interactive learning tools
- When rolling out the Personalized breast cancer risk assessment initiative, would it be possible to have more connections with the Canadian Breast Screening Network, i.e. online education/webinars?		Collaborate with other Canadian breast cancer initiatives
- Terrific idea. Most prevention medicine is NOT remunerated in NL. There should be a fee code for this. The 1st two visits will take more than 10 minutes	1	Consider adding a fee code for medical consultation targeting prevention
- More education would be great!		Need more information about the risks and benefits
- Nurses need more education on screenings		Enhance nurses' training
- For the first set of questions, I think women needs to understand benefits and harms of screening in addition to their risk of breast cancer - therefore I disagree with recommending breast screening for all.		Need more information about the risks and benefits

Note 1. Respondents' comments were only lightly edited for obvious spelling mistakes.

Note 2. Definition of acronyms in the language of the comment: ARQ=agence de revenue du Québec; ATCD=antécédent; App=application; BAC=baccalauréat; CAD=canadian; CISSS=centre intégré de santé et de services sociaux; CIUSSS= centre intégré universitaire de santé et de services sociaux; CME=continuing medical education; GMF=groupe de médecine familiale; IPSPL=infirmière praticienne spécialisée de première ligne; Mammo=mammographie; MD=médecin; MRI=magnetic resonance imaging; NL=Newfoundland and Labrador; NP=nurse practitioner; OMS=Organisation mondiale de la santé; Pts=patients; PQDCS=programme Québécois de dépistage du cancer du sein; RDV=rendez-vous.
